# Valorisation of pectin-rich agro-industrial residues by yeasts: potential and challenges

**DOI:** 10.1007/s00253-020-10697-7

**Published:** 2020-05-31

**Authors:** Luís C. Martins, Catarina C. Monteiro, Paula M. Semedo, Isabel Sá-Correia

**Affiliations:** 1 iBB - Institute for Bioengineering and Biosciences, Instituto Superior Técnico, Universidade de Lisboa, Lisbon, Portugal; 2 Department of Bioengineering, Instituto Superior Técnico, Universidade de Lisboa, Lisbon, Portugal

**Keywords:** Pectin-rich agro-industrial residues, Non-conventional yeasts, Bioconversion, Metabolic engineering, Biorefinery, Circular bioeconomy

## Abstract

Pectin-rich agro-industrial residues are feedstocks with potential for sustainable biorefineries. They are generated in high amounts worldwide from the industrial processing of fruits and vegetables. The challenges posed to the industrial implementation of efficient bioprocesses are however manyfold and thoroughly discussed in this review paper, mainly at the biological level. The most important yeast cell factory platform for advanced biorefineries is currently *Saccharomyces cerevisiae*, but this yeast species cannot naturally catabolise the main sugars present in pectin-rich agro-industrial residues hydrolysates, in particular d-galacturonic acid and l-arabinose. However, there are non-*Saccharomyces* species (non-conventional yeasts) considered advantageous alternatives whenever they can express highly interesting metabolic pathways, natively assimilate a wider range of carbon sources or exhibit higher tolerance to relevant bioprocess-related stresses. For this reason, the interest in non-conventional yeasts for biomass-based biorefineries is gaining momentum. This review paper focuses on the valorisation of pectin-rich residues by exploring the potential of yeasts that exhibit vast metabolic versatility for the efficient use of the carbon substrates present in their hydrolysates and high robustness to cope with the multiple stresses encountered. The major challenges and the progresses made related with the isolation, selection, sugar catabolism, metabolic engineering and use of non-conventional yeasts and *S. cerevisiae*-derived strains for the bioconversion of pectin-rich residue hydrolysates are discussed. The reported examples of value-added products synthesised by different yeasts using pectin-rich residues are reviewed.

**Table:** 

**Key Points** *• Review of the challenges and progresses made on the bioconversion of pectin-rich residues by yeasts.* *• Catabolic pathways for the main carbon sources present in pectin-rich residues hydrolysates.* *• Multiple stresses with potential to affect bioconversion productivity.* *• Yeast metabolic engineering to improve pectin-rich residues bioconversion.*

**Key Points**

*• Review of the challenges and progresses made on the bioconversion of pectin-rich residues by yeasts.*

*• Catabolic pathways for the main carbon sources present in pectin-rich residues hydrolysates.*

*• Multiple stresses with potential to affect bioconversion productivity.*

*• Yeast metabolic engineering to improve pectin-rich residues bioconversion.*

**Figure:**
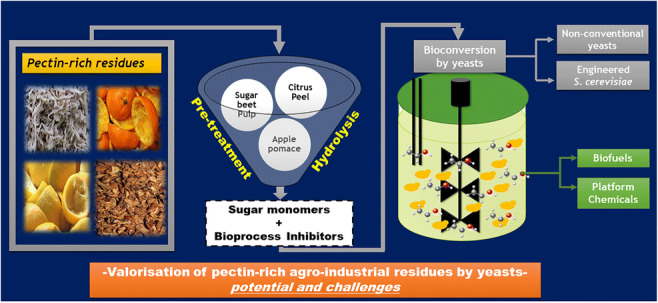
Graphical abstract

Graphical abstract

## Introduction

Agro-industrial residues are currently in the spotlight of research and development activities worldwide; they are raw materials for the biotechnology industry, as renewable sources of carbon, nitrogen and other nutrients for microbial growth and metabolite production (Cherubini 2010; Liguori and Faraco 2016; Liu et al. 2016; Dahiya et al. 2018). The utilisation of organic waste residues as substrates to produce added-value products is environmentally friendly strategies by saving and reutilizing resources. The implementation of a circular bioeconomy based on microorganisms, in particular non-conventional (non*-Saccharomyces*) yeast strains with metabolic versatility and tolerance to bioprocesses-related stresses, is an important societal challenge (Leandro et al. 2006; Fletcher et al. 2016; Cristobal-Sarramian and Atzmuller 2018; Zuin et al. 2018; Rebello et al. 2018; Nielsen 2019).

Agro-industrial residues were derived from sugary materials (e.g. sugar beet, sugarcane or fruits and vegetables) and starchy feedstocks (e.g. wheat, corn, rice or potatoes) and lignocellulosic substrates (e.g. wood, straw and grasses) (Balat 2011). Pectin-rich agricultural residues and agro-food industry residues are potential feedstocks for the production of biofuels and other relevant bioproducts (Schmitz et al. 2019). Currently, a large fraction of the pectin-rich residues (e.g. sugar beet pulp and citrus peel) are dried for further use as cattle feed or put in landfills for soil improvement, although it is desirable to find new ways to convert these residues into renewable chemicals using natural or engineered microbes (Richard and Hilditch 2009; Ajila et al. 2012). The residues with the highest pectin content (sugar beet pulp, citrus peels, and apple pomace) are accumulated in high amounts worldwide from the sugar industry or the industrial processing of fruits and vegetables (Peters 2006; Balat 2011). These residues are partially pre-treated during sugar (from sugar beets) and juice (from fruits) extraction and have low lignin content which facilitates processing (Berlowska et al. 2018). Despite the difficulties inherent to the high variability of these feedstocks due to diverse geographical distribution and seasonality, they are cheap and abundant (Peters 2006; Balat 2011). However, they are interesting feedstocks for microbial fermentations, as the enzymatic hydrolysis of their component polysaccharides can be economically accomplished to yield fermentable neutral sugars (hexoses and pentoses) and d-galacturonic acid (d-GalA) (Leijdekkers et al. 2013; Cárdenas-Fernández et al. 2017; de la Torre et al. 2019).

*Saccharomyces cerevisiae* is currently, and by far, the most important yeast cell factory in the biotechnology industry and the major cell factory platform for the production of bioethanol and other biofuels and chemicals in advanced biorefineries (Satyanarayana and Kunze 2009; de Jong et al. 2012; Hong and Nielsen 2012; Nielsen 2019). Endogenously, *S. cerevisiae* can only use a very limited range of carbon sources. For this reason, genetically modified strains have been developed to also utilise pentoses and d-galacturonic acid for synthesis of novel compounds (Hong and Nielsen 2012; Benz et al. 2014; Biz et al. 2016; Yaguchi et al. 2018; Rebello et al. 2018; Protzko et al. 2018; Nielsen 2019). However, there are non-conventional species considered advantageous alternatives to *S. cerevisiae* since they can express highly interesting metabolic pathways (Rebello et al. 2018), efficiently assimilate a wider range of carbon sources (Do et al. 2019) or exhibit higher tolerance to relevant bioprocess-related stresses, such as the presence of a wide range of inhibitory compounds and supraoptimal temperatures (Radecka et al. 2015; Kręgiel et al. 2017; Mukherjee et al. 2017). Several non-conventional yeast species are capable of producing high concentrations of sugar alcohols (namely xylitol and arabitol) (Schirmer-Michel et al. 2009; Loman et al. 2018), lipids and single-cell oils for food or energy applications (Ratledge 2010; Taskin et al. 2016; Anschau 2017; Hicks et al. 2020), enzymes (Serrat et al. 2004; Saravanakumar et al. 2009; Sahota and Kaur 2015) and pigments (Buzzini and Martini 2000; Aksu and Eren 2005) among other added-value compounds. It should be noted that different yeast species, and even strains, significantly differ in the products synthesised and in their production rates and yields (Rodríguez Madrera et al. 2015; van Dijk et al. 2019). Non-conventional yeasts have recently been in the focus of active and relevant research, their genome sequences are being released and suitable genetic engineering tools are either available or being developed for different purposes (Mira et al. 2014; Palma et al. 2017; Nambu-Nishida et al. 2017; Lee et al. 2018; Cai et al. 2019; Protzko et al. 2019; among several other examples). Thus, it is expected that, in the near future, the currently accepted designation of “non-conventional yeast” will no longer be adequate and non-*Saccharomyces* strains will successfully be used in the industry (Johnson 2013a; Radecka et al. 2015; Kręgiel et al. 2017; Siripong et al. 2018). This review paper presents relevant results and discusses the potential and the current challenges of the use of yeasts for the valorisation of pectin-rich agro-industrial residues.

### Pectin-rich agro-industrial residues as feedstocks for biotechnology

#### Pectin structure and pectin-rich biomasses

**Fig. 1:**
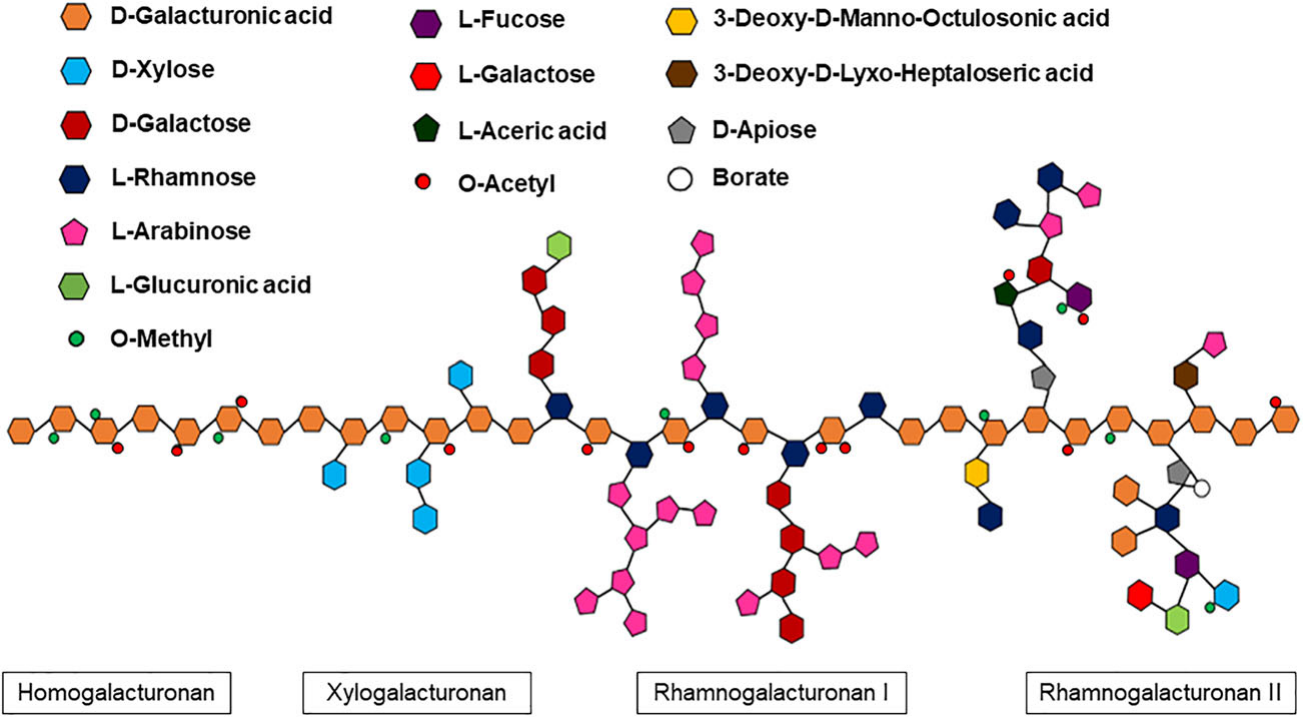
Schematic representation of the chemical structure of four pectic polysaccharides: homogalacturonan (HG), substituted HG xylogalacturonan (XGA) and rhamnogalacturonan I and II (RG-I and RG-II), based on (Mohnen 2008)

Pectin is a family of complex heteropolysaccharides and a structural component of plant cell walls (Mohnen 2008). Pectin is composed of a linear chain of α-1,4 linked d-galacturonic acid (d-GalA) molecules which represent about 70% of total weight in a homogalacturonan polymer. There are three major pectin polymers: homogalacturonan (HG), rhamnogalacturonan I (RG-I) and rhamnogalacturonan II (RG-II) (Fig. 1). More complex pectin structures, such as rhamnogalacturonan I and II, have side chains composed by neutral sugars that include l-rhamnose, l-arabinose, d-xylose, d-galactose, l-fucose and d-glucose, among others (Sakai et al. 1993). These sugars are linked to d-galacturonic acid by β-1,2 and β-1,4 glycosidic linkages (Fig. 1) (Jayani et al. 2005; Mohnen 2008). Moreover, d-galacturonic acid (d-GalA) residues can be methyl-esterified at the C6 carboxyl group and/or O-acetylated at C-2 or C-3 and neutralised by ions, like sodium, calcium or ammonium (Sakai et al. 1993; Jayani et al. 2005).

**Fig. 2:**
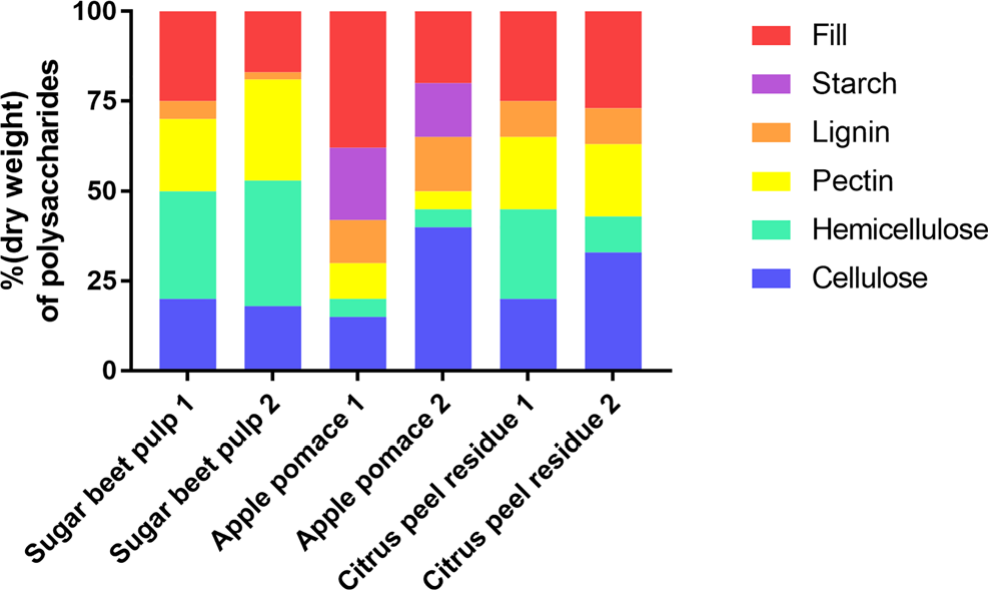
Dry-weight composition of pectin-rich residues, in particular sugar beet pulp 1 (Berlowska et al. 2018) and 2 (Edwards and Doran-Peterson 2012), apple pomace 1 (Grohmann and Bothast 1994) and 2 (Bhushan et al. 2008) and citrus peel 1 (Zhou et al. 2008) and 2 (John et al. 2017)

Pectin-rich biomasses, in particular the agro-food residues left after fruit or vegetable processing for juice or sugar production (e.g. apple pomace, citrus waste, and sugar beet pulp), are abundant and widely underused bioresources (Zhou et al. 2008; Mohnen 2008). Although most food waste streams contain pectin too, the residues mentioned above exhibit the highest pectin content with pectin concentrations ranging from 12 to 35% of the biomass dry weight (Müller-Maatsch et al. 2016). The low lignin content of these processed wastes is an interesting trait because lignin can impair with the enzymatic degradation of cellulose and hemicellulose, and its monomers cannot be used as carbon sources so far (Guo et al. 2009). Lignin, the most recalcitrant cell wall material, can be combusted and converted into electricity and heat (Limayem and Ricke 2012). The composition of agro-food residues and the bioavailability of their various polysaccharide fractions are highly dependent on natural variation, husbandry practices, fruit maturity and post-harvest management (Grohmann and Bothast 1994). The apparent high variability of the different pectin-rich biomasses regarding the dry-weight composition in pectin and other polysaccharides is shown in Fig. 2. Determining pectin in biomass quantitatively is actually quite challenging and the differences detected may simply result from the use of different analytic methods and sub-optimal techniques (Quemener et al. 1993; Kühnel 2011; Wikiera et al. 2015).

#### Pectin-rich biomass processing and composition of the resulting hydrolysates

The bioconversion of pectin-rich agro-industrial residues requires pre-treatment step(s) before microbial utilisation in order to avoid the recalcitrant material and to increase surface area to facilitate and enhance the hydrolysis step (Limayem and Ricke 2012). After pre-treatment, the enzymatic or acidic hydrolysis of cellulose, hemicellulose and pectin structures allow the release of monomeric sugars (saccharification) that will subsequently be converted into ethanol and/or other bioproducts by yeasts. Filamentous fungi, in particular *Aspergillus* sp., *Trichoderma reesei* and *Neurospora crassa*, naturally have degrading machinery consisting in hydrolytic and oxidative enzymes which play an important role in plant biomass degradation (Schmitz et al. 2019). A recent review details the enzyme repertoire of filamentous fungi and their specific transcriptional regulation patterns for efficient biomass degradation (Benocci et al. 2017).

Remarkably, the sugar composition of the hydrolysates obtained from the same pectin-rich agro-industrial residue is highly dependent on the pre-treatment and enzyme hydrolysis conditions used (Table 1) reducing the reproducibility of the hydrolysis process (Merz et al. 2016).

**Table 1:** Composition of pectin-rich agro-industrial residues hydrolysates depending on their pre-treatment and hydrolysis

Feedstock	Pre-treatment	Hydrolysis	Sugar composition after hydrolysis (g/100 g matter)	Reference
Sugar beet pulp	Steam explosion at 152 °C to 175.5 °C and 4 to 8 bar pressure	(Soluble fraction) Acid hydrolysis: 72%H_2_SO_4_ for 1 h at 30 °C and 150 rpm	Glucose 26	Hamley-Bennett et al. (2016)
			Arabinose 24	
			Xylose 1.6	
			Rhamnose 2.4	
			Galactose 6	
			Galacturonic acid 14	
		(insoluble fraction) Enzyme hydrolysis: 0.5 mg cellulase/g glucan 50 °C with shaking for 24 h	Glucose 10	
			Arabinose 0.4	
			Xylose 0.3	
			Rhamnose 0.1	
			Galactose 0.1	
			Galacturonic acid <0.1	
	Not used	Enzyme hydrolysis: Viscozyme and Ultraflo Max (Novozymes) treatment	Total of reducing sugars 6.6	Berlowska et al. (2017)
Apple pomace	15 g/L sulphuric acid for 16 min at 91 °C with laccase 100 units/L at 30 °C for 12 h at 90 rpm	Enzyme hydrolysis: Viscozyme and Celluclast (0.5 μL/mL, 0.038 mg/mL) together with Novozyme188 (0.05 μL/mL, 0.0024 mg/mL)	Galacturonic acid 33	Gama et al. (2015)
			Glucose 21	
			Arabinose 17	
			Galactose 5	
	Not used	Acid hydrolysis 1.5 g sulphuric acid/100 mL, 91 °C reaction temperature during 16 min	18.2 g of glucose and fructose/100 g dry matter	Parmar and Rupasinghe (2012)
	Not used	Acid hydrolysis 72% sulphuric acid for 45 min at room temperature and diluted with distilled water to 4% sulphuric acid, followed by autoclaving for 1 h at 121 °C	Galacturonic acid (*not quantified*)	Choi et al. (2015)
			Glucose 25	
			Fructose 24	
			Arabinose 6	
			Sucrose 9	
			Galactose 4	
			Xylose 6	
			Rhamnose 2	
	Not used	Acid hydrolysis 2 M Trifluoroacetic acid for 2 h at 100 °C with constant shaking	*Rhamnose 0.5*	Wikiera et al. (2015)
			*Arabinose 8*	
			*Glucose 12*	
			*Galactose 4*	
			*Xylose 4*	
			*Mannose 0.7*	
	Acid hydrolysis 0.2 M Trifluoroacetic acid for 72 h at 80 °C with constant shaking	Enzyme hydrolysis: Viscozyme (25 μL) incubated at 50 °C during 24 h with constant shaking	*Rhamnose 0.4*	
			*Arabinose 7*	
			*Glucose 11*	
			*Galactose 4*	
			*Xylose 4*	
			*Mannose 0.3*	
Citrus peel	80% v/v ethanol for 20 min, filtered on a sintered glass, and dried at 40 °C for 72 h	Acid hydrolysis: 0.05 M hydrochloric acid at 85 °C	Galacturonic acid 15	Yapo et al. (2007a)
			Arabinose 4	
			Galactose 1	
			Glucose 1	
			Rhamnose 0.5	
	Not used	Acid hydrolysis 72% sulphuric acid for 45 min at room temperature and diluted with distilled water to 4% sulphuric acid, followed by autoclaving for 1 h at 121 °C	Orange peel	Choi et al. (2015)
			Galacturonic acid (*not quantified*)	
			Glucose 36	
			Fructose 12	
			Arabinose 6	
			Sucrose 5.6	
			Galactose 3	
			Xylose 2	
			Rhamnose 2	
			Lemon peel	
			Galacturonic acid (*not quantified*)	
			Glucose 27	
			Fructose 3	
			Arabinose 5	
			Sucrose 0	
			Galactose 5	
			Xylose 3	
			Rhamnose 2	
	Steam explosion at 150 °C for 10 min and 15 kg/cm pressure	Pectinase, xylanase (5 mg/g dry matter) and β-glucosidase (2 mg/g dry matter) cocktail at 45 °C for 24 h	Galacturonic acid (*not quantified*)	Choi et al. (2013)
			Glucose 45	
			Fructose 18	
			Arabinose 3	
			Galactose 2	

Pectin-rich biomass hydrolysates may also include growth inhibitory compounds, such as weak acids, furan derivatives and phenolic compounds generated during pre-treatment and acid hydrolysis of pectin-rich materials (Palmqvist and Hahn-Hägerdal 2000). Acetic acid and methanol are potential growth inhibitors that are likely to be present (Vendruscolo et al. 2008; Günan Yücel and Aksu 2015; Berlowska et al. 2018). These compounds have potential to affect yeast growth, fermentation kinetics and metabolite production yields, (dos Santos and Sá-Correia 2015; Cunha et al. 2019). Although, the individual toxicity of some of these compounds can be relatively low, their combined toxic effects can be additive or even synergistic (Palmqvist and Hahn-Hägerdal 2000; Teixeira et al. 2011). The average degree of methylation and acetylation of diverse pectin-rich residues is different with sugar beet exhibiting the highest acetylation degree (Table 2). Other potentially critical inhibitors are heavy metals and pesticides. They have also been detected in pectin-rich residues, mainly due to the geochemical cycles and human activities, such as intensive agriculture, waste treatment and disposal and transportation (Legrand 2005; Skrbic et al. 2010; Mukherjee et al. 2017).

**Table 2:** Percentage (of total dry matter) of acetylation and methylation of different pectin-rich materials

Pectin substrate	Acetylation (%)	Methylation (%)	References
Citrus fruits (orange, lime, lemon)	3	60–80	Sakai et al. (1993); Yapo et al. (2007a); Williams (2011)
Apple	4	80	Sakai et al. (1993); Williams (2011)
Sugar beet	10–20	Up to 60	Sakai et al. (1993); Yapo et al. (2007b); Williams (2011)

### Yeast metabolism of sugar monomers present in pectin-rich hydrolysates

#### The challenges

The efficient utilisation by yeasts of the mixtures of sugar monomers present in hydrolysates derived from pectin-rich residues is essential for their biotechnological valorisation. Sugar beet pulp and citrus peel hydrolysates contain predominantly the neutral sugars l-arabinose, d-glucose and d-galactose and the acidic sugar d-galacturonic acid (Micard et al. 1996; Berlowska et al. 2018). This means that the convenient yeast species/strains to be used should be able to rapidly and efficiently catabolise all the sugars present (Du et al. 2019).

The presence and simultaneous use of several sugars in pectin hydrolysates is an important challenge also due to carbon catabolite repression (CCR) regulation (Kayikci and Nielsen 2015; Gao et al. 2019). This regulation mechanism limits the efficient utilisation of multiple carbon substrates in biotechnological processes like those developed for the valorisation of pectin-rich residues. In fact, the uptake of secondary carbon sources (e.g. l-arabinose, d-galacturonic acid, d-xylose) is inhibited in the presence of the preferred substrate (d-glucose), prolonging fermentation time as the result of sequential, rather than the simultaneous, use of the carbon sources (Huisjes et al. 2012; Wu et al. 2016; Yaguchi et al. 2018; Lane et al. 2018). *S. cerevisiae* has a highly complex and still not fully understood network of signals and regulations, through (de)phosphorylation mechanisms depending on the presence of d-glucose in the medium which have been on the focus of extensive review papers (Gancedo 1992; Conrad et al. 2014; Kayikci and Nielsen 2015).

**Fig. 3:**
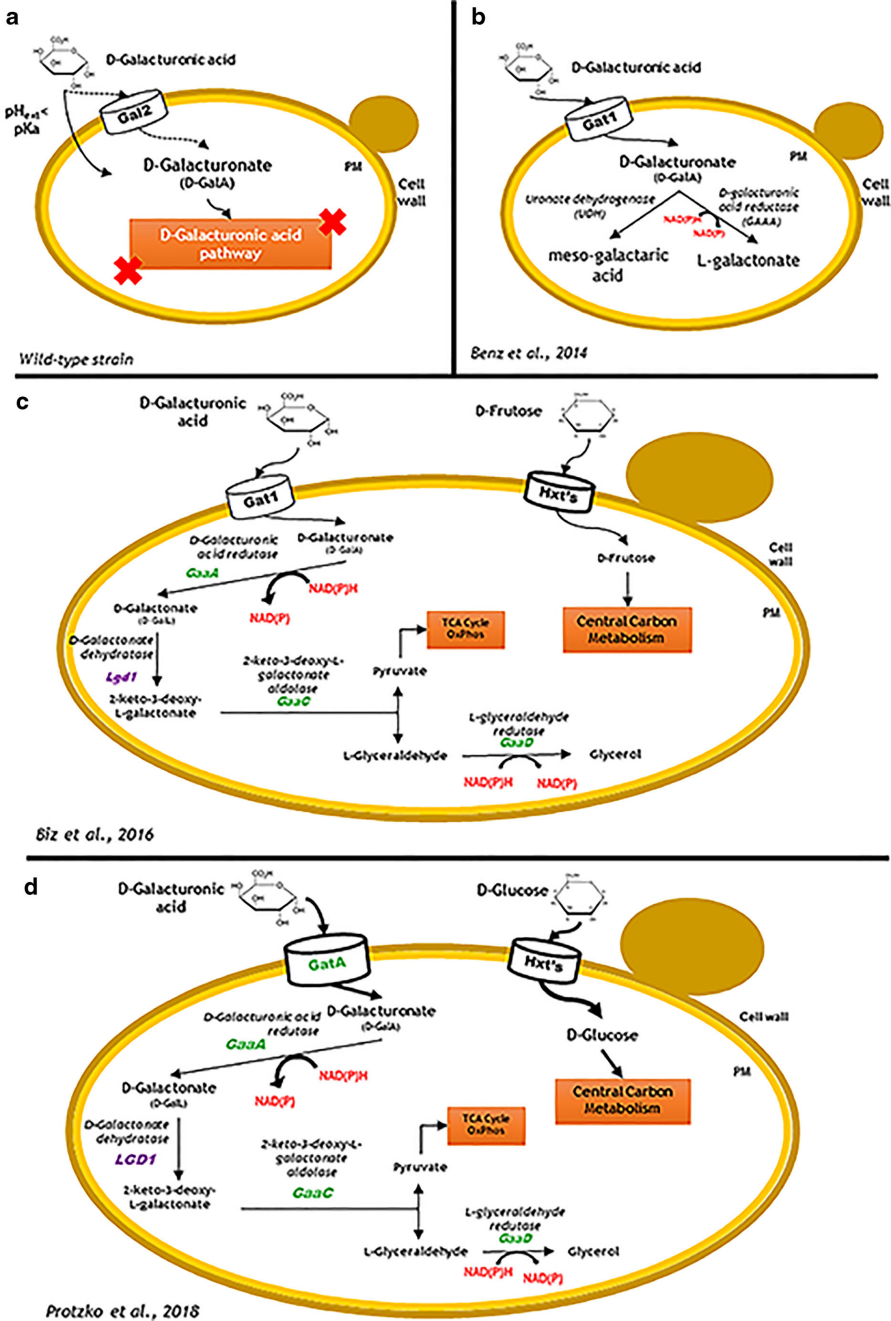
Schematic representation of *S. cerevisiae* strains (wild type and genetically engineered with heterologous d-galacturonic acid degradation pathways. **a***S. cerevisiae* wild-type strain showing the basal natural uptake of d-galacturonic acid by Gal2p transporter and passive diffusion of the undissociated form through plasma membrane (PM). **b** Engineered *S. cerevisiae* strains expressing d-GalA membrane transporter Gat1 from *Neurospora crassa* and the uronate dehydrogenase (UDH) from *Agrobacterium tumefaciens* and d-galacturonic acid reductase (GAAA) from *Aspergillus niger* to convert d-GalA into the metabolites meso-galactaric acid and *l*-galactonate, (Benz et al. 2014). **c** Engineered *S. cerevisiae* strain with d-galacturonic acid plasma membrane transporters from *N. crassa* (GAT1) and enzymes of the d-GalA catabolic pathway GaaA, GaaB, GaaC and GaaD from *A. niger* (in green) and LGD1 from *Trichoderma reesei* (in purple); d-Fructose was used as co-substrate (Biz et al. 2016). **d** Engineered *S. cerevisiae* strains with the non-glucose repressible plasma membrane d-galacturonic acid transporter GatA from *A. niger* (GATA) and d-GalA catabolic pathway as in **c**); d-glucose was used as co-substrate (Protzko et al. 2018)

**Fig. 4:**
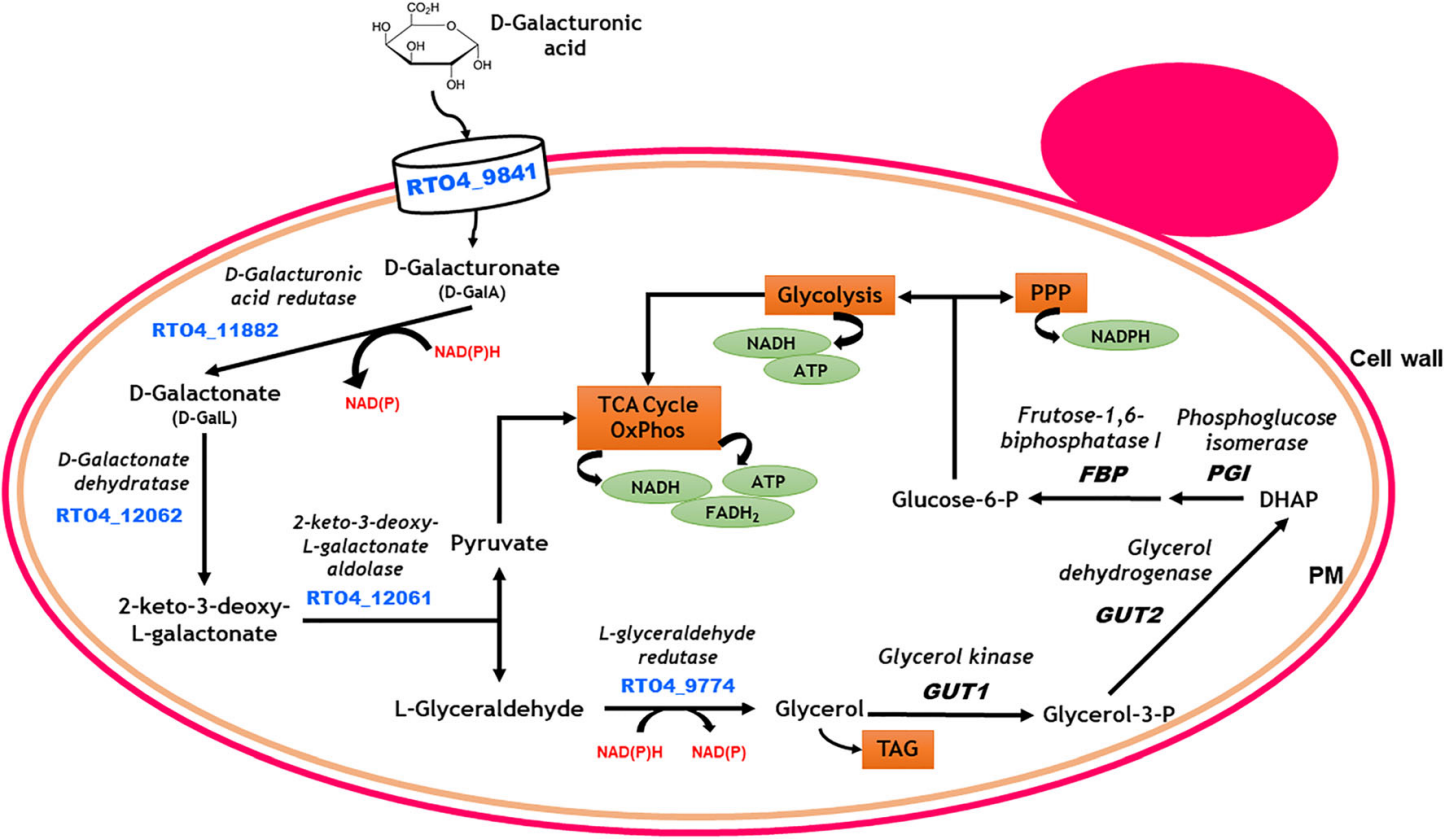
Schematic representation of the d-galacturonic acid catabolic pathway proposed for *Rhodosporidium toruloides* IFO0880. The genes *GUT1*, *GUT2*, *FBP* and *PGI* belong to central metabolism. TAG, triacylglycerol; PPP, pentose phosphate pathway (based on (Protzko et al. 2019)

**Fig. 5:**
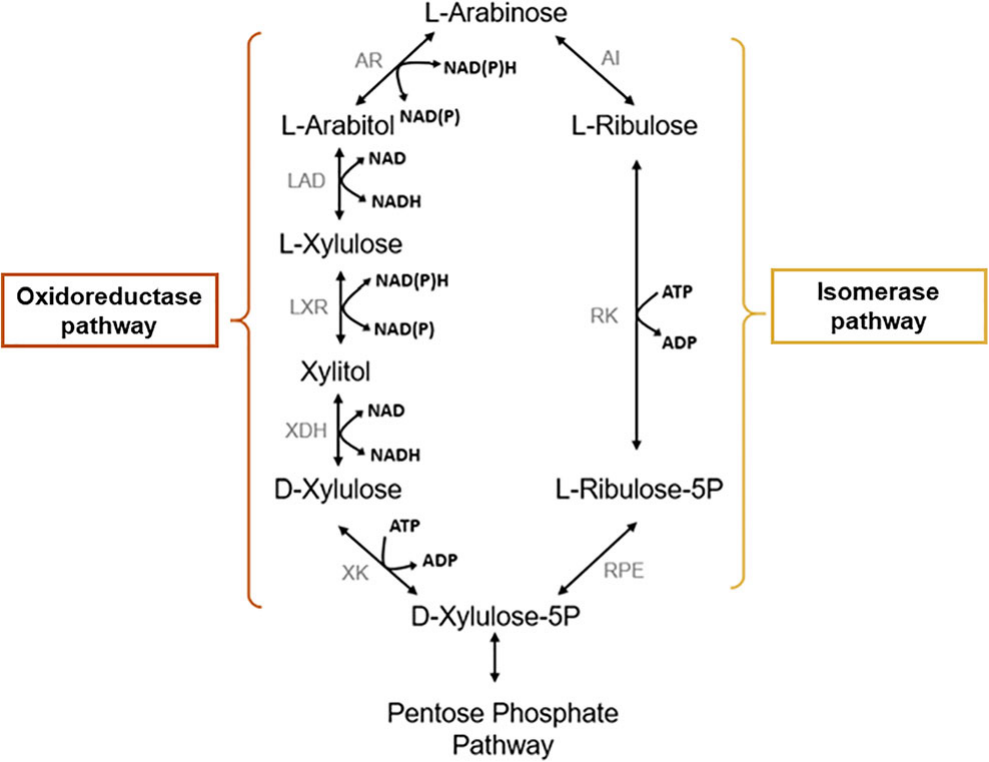
Schematic representation of the initial steps of arabinose metabolism in fungi (the oxidoreductase pathway) or in bacteria (the isomerase pathway). XK, d-xylulose kinase; AI, l-arabinose isomerase; RK, *l*-ribulokinase; RPE, *l*-ribulose-5-phosphate 4-epimerase; XDH, xylitol dehydrogenase; AR, *l*-arabinose reductase; LAD, *l*-arabitol 4-dehydrogenase; LXR, *l*-xylulose reductase (adapted from Fonseca et al., 2007)

Moreover, pectin-rich hydrolysates contain a significant amount of d-galacturonic acid that is neither naturally used by *S. cerevisiae* nor by other relevant yeast species, such as *Kluyveromyces marxianus*, *Yarrowia lipolytica*, *Pichia stipitis*, among others. Recent efforts have been reported in order to genetically engineer *S. cerevisiae* to efficiently express the d-galacturonic acid catabolic pathway (Benz et al. 2014; Zhang et al. 2015; Nielsen and Keasling 2016; Matsubara et al. 2016; Biz et al. 2016; Kalia and Saini 2017; Lian et al. 2018; Protzko et al. 2018; Jeong et al. 2020). Moreover, since pectin-rich hydrolysates have significant amounts of l-arabinose, efforts have also addressed the expression of this pentose-fermentative pathway in *S. cerevisiae* strains (Wisselink et al. 2007; Ye et al. 2019). The pathways involved in the catabolism of d-galacturonic acid and l-arabinose, the main sugars released from pectin-rich feedstocks hydrolysis, are detailed below (Figs. 3, 4, 5). d-galacturonic acid catabolic pathway is emphasised because this acid sugar catabolism is currently the big challenge for which there is relevant recent literature. Non-conventional yeast species/strains reported in the scientific literature as naturally capable of such catabolism are also referred. Due to space limitations and the more-established catabolism of most of the other sugars present or their marginal concentrations in the hydrolysates, the corresponding pathways are not described here.

#### d-galacturonic acid pathways from fungi, expressed in *S. cerevisiae*, and from the oleaginous yeast *Rhodosporidium toruloides*

d-galacturonic acid is not catabolised by the yeast *S. cerevisiae* that misses the catabolic pathway (Fig. 3a). Moreover, as an acid sugar, d-galacturonic acid is more oxidised than the neutral hexose and pentose sugars. This means that its metabolism is not redox neutral as glucose metabolism and the fermentation of d-galacturonic acid requires more NADPH cofactor molecules to produce ethanol (Richard and Hilditch 2009). The d-galacturonic acid plasma membrane transporter Gat1 from *N. crassa* was identified and characterised and the encoding gene *GAT1* successfully expressed in *S. cerevisiae* allowing the increased uptake of d-galacturonic acid in this yeast cell factory (Benz et al. 2014) (Fig. 3b). In fact, d-galacturonic acid uptake is poorly performed when mediated by the native Gal2 or other hexose transporters (Huisjes et al. 2012; Benz et al. 2014; Biz et al. 2016), even though d-galacturonic acid was shown to be taken up rapidly by *S. cerevisiae* (Souffriau et al. 2012). With the co-expression in yeast of a d-galacturonic acid reductase (from the filamentous fungus *Aspergillus niger*) or a uronate dehydrogenase (from the bacterium *Agrobacterium tumefaciens* involved in plant infection), a transporter-dependent conversion of d-galacturonic acid towards more reduced (l-galactonate) or oxidised (*meso*-galactaric acid) downstream metabolites was also demonstrated (Fig. 3b) (Benz et al. 2014). This heterologous co-expression, although highly relevant as proof of concept, missed the expression of the complete d-galacturonic catabolic pathway for the full catabolisation of this acid sugar.

**Fig. 6:**
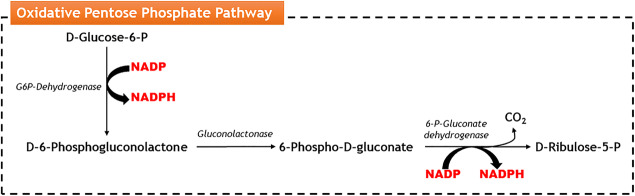
Schematic representation of the oxidative pentose phosphate pathway (Wamelink et al. 2008)

Therefore, several efforts have been made envisaging the development of a genetically engineered *S. cerevisiae* strain capable of efficiently using d-galacturonic acid from pectin-rich hydrolysates. For this purpose, the genes *GAAA*, *GAAC*, *GAAD* encoding d-galacturonic acid reductase, 2-keto-3-deoxy- l-galactonate aldolase, respectively, from *A. niger* and the gene *LGD1* encoding d-galactonate dehydratase from *T. reesei* were successfully expressed in *S. cerevisiae* (Fig. 3c) (Biz et al. 2016). The entire d-galacturonic acid catabolic pathway from filamentous fungi comprises two NADPH-dependent enzymes: the d-galacturonate reductase and the l-glyceraldehyde reductase, for the catabolisation of d-galacturonic acid into glycerol (Biz et al. 2016) (Fig. 3c) leading to intracellular cofactor imbalance. For the efficient functioning of d-galacturonic acid catabolic pathway from filamentous fungi in *S. cerevisiae*, the pathway has to be coupled with NADPH regeneration steps which can be achieved through the operation of the oxidative pentose phosphate pathway (PPP). The oxidative PPP converts d-glucose-6-P into d-ribulose-5-P and CO_2_ with the simultaneous reduction reaction of two molecules of NADP to NADPH (Fig. 6) (Wamelink et al. 2008). This cofactor regeneration may enable the catabolisation of d-galacturonic acid in engineered *S. cerevisiae* strains. However, the use of co-substrates such as d-fructose or d-glucose is required although their presence leads to the delay of d-galacturonic acid catabolisation (Benz et al. 2014; Biz et al. 2016). The successful heterologous expression of the *A. niger*d-galacturonic acid transporter *GatA* in *S. cerevisiae* allowed the co-uptake of d-galacturonic acid and d-glucose which could also facilitate the regeneration of redox cofactors needed for full conversion of d-galacturonic acid (Fig. 3d) (Protzko et al. 2018). A more recent study reported the expression of the efficient previously described fungal d-galacturonic acid catabolic pathway in a pentose-fermenting *S. cerevisiae* strain by the expression of a pentose (d-xylose and l-arabinose) catabolic pathway including genes from *Pichia stipitis* and *Ambrosiozyma monospora*, both natural pentose-fermentative yeasts (Jeong et al. 2020). Additionally, the authors made a double deletion from genes *PHO13* (involved in phosphatase regulation) and *ALD6* (a cytosolic aldehyde dehydrogenase required for conversion of acetaldehyde to acetate). All these genetic modifications enabled the co-consumption of more than 10 g/L of d-galacturonic acid with l-arabinose and d-xylose (Ye et al. 2019; Jeong et al. 2020).

The genome-wide and enzymatic analysis of the basidiomycete red oleaginous yeast *Rhodosporidium toruloides* (also known as *Rhodotorula toruloides*) IFO0880 revealed an efficient d-galacturonic acid metabolism, with highly active enzymes (Fig. 4), suggesting this strain as a potential industrial platform for biodiesel and carotenoid biosynthesis from pectin-rich hydrolysates (Sitepu et al. 2014; Spagnuolo et al. 2019; Protzko et al. 2019). The d-galacturonic acid metabolic pathway of *R. toruloides* was found to be similar to the *T. reesei* pathway, being the catabolic enzymes highly induced by d-galacturonic acid (Protzko et al. 2019). Moreover, *R. toruloides* IFO0880 was found to co-utilise d-galacturonic acid in the presence of either d-glucose or d-xylose. The final product of d-galacturonic acid catabolic pathway is glycerol that has to be used for cofactor regeneration through the oxidative pentose phosphate pathway. The study performed in *R. toruloides* IFO0880 also showed that the genes *GUT1*, encoding a glycerol kinase, and *GUT2*, encoding a mitochondrial glycerol 3-phosphate dehydrogenase, involved in glycerol metabolism and induced in presence of d-galacturonic acid, enabled d-galacturonic acid conversion into glycerol without the need of an additional carbon source. This study proposed that the glycerol produced could be converted into glucose-6-phosphate and, through the oxidative pentose phosphate pathway, the cofactors used in d-galacturonic acid catabolisation would be regenerated (Protzko et al. 2019). Different routes of glycerol catabolic pathways have been described in yeasts, using NAD^+^- or NADP+-dependent enzymes, balancing the intracellular redox power and enabling growth in respirable carbon sources (Klein et al. 2017).

#### l-arabinose metabolism

l-arabinose is a five-carbon sugar and, unlike other pentoses that naturally occur in the d-form such as d-xylose, l-arabinose is more common than d-arabinose in nature. Arabinose catabolic pathways include the oxidoreductase (fungal) and the isomerase (bacterial) pathways (Fig. 5). In both pathways, l-arabinose is converted into d-xylulose-5-phosphate, which is metabolised by the non-oxidative phase of the pentose phosphate pathway.

In the fungal pathway, l-arabinose reductase (AR) prefers NADPH as cofactor, whereas the sugar alcohol dehydrogenases (LAD and XDH) are strictly dependent on NAD (Seiboth and Metz 2011). Under low oxygen conditions, the availability of NAD is limited, which may cause an accumulation of l-arabitol (Loman et al. 2018). Furthermore, l-arabinose can be converted into xylitol, the common denominator between the catabolic pathways of l-arabinose and d-xylose (Fig. 5). Due to their partially overlapping pathways, there is a strong correlation between the utilisation of these two pentoses in yeasts (Seiboth and Metz 2011). The introduction of a reconstructed fungal l-arabinose oxidoredutase pathway (from *T. reesei* and *A. monospora* strains) into *S. cerevisiae* allowed l-arabinose utilisation and the production of substantial amounts of l-arabitol due to the severe redox imbalance resulting from the utilisation of NADPH in the reduction step catalysed by l-xylulose reductase (LXR) (Bettiga et al. 2009). In fact, l-xylulose reductase from *A. monospora* is NADH-dependent enzyme, contrarily to most fungi which are NADPH-dependent for this specific enzyme. However, NADH is produced in the oxidation reactions catalysed by l-arabitol-4-dehydrogenase (LAD) and xylitol dehydrogenase (XDH) improving intracellular redox balance (Bettiga et al. 2009).

Although l-arabinose fermentation by yeasts was thought to be unfeasible, several yeast species have been identified as capable of producing ethanol from l-arabinose, in particular *Candida auringiensis*, *Candida succiphila*, *A. monospora*, *Candida* sp. (YB-2248) (Dien et al. 1996), and *Meyerozyma guilliermondii* (Martini et al. 2016). Moreover, the successful engineering of *S. cerevisiae* to ferment l-arabinose, by expressing the l-arabinose isomerase pathway of the bacterial species of *Lactobacillus plantarum* (Fig. 5) and overexpressing the *S. cerevisiae* genes encoding the enzymes of the non-oxidative pentose phosphate pathway, along with extensive evolutionary engineering, resulted in ethanol production (0.43 g g^−1^) from l-arabinose during anaerobic growth (Wisselink et al. 2007). To increase l-arabinose fermentation rates, potential l-arabinose transporters have been identified and overexpressed in *S. cerevisiae*. For example, the overexpression of *S. cerevisiae* Gal2 led to the increase of l-arabinose fermentation rate (Becker and Boles 2003). However, this endogenous *S. cerevisiae* hexose transporter not only exhibits very low affinities towards pentoses but is also strongly inhibited by glucose (Gao et al. 2019). The expression of heterologous transporters with higher affinities for arabinose over glucose, in particular of Stp2 from *Arabidopsis thaliana* and AraT from *Scheffersomyces stipitis*, led to the improvement of l-arabinose fermentation, in anaerobiosis, especially at low l-arabinose concentrations. However, l-arabinose uptake through these two transporters is also inhibited by the presence of glucose (Subtil and Boles 2011).

### Toxicity and possible metabolisation of compounds likely present in pectin-rich biomass hydrolysates

#### Multiple chemical stresses likely affecting pectin-rich biomass bioconversion

It is likely that pectin-rich residues may include variable levels of toxic compounds. Frequently, their concentrations are not always known or even considered, but these compounds may have a potential combined inhibitory effect for yeast growth and metabolism, acting in conjunction or synergistically. In particular, since pectin structures are acetylated and methyl-esterified in different positions of the d-galacturonic acid molecule, this biomass hydrolysis releases acetic acid and methanol that accumulate in the hydrolysate. The potential role of these compounds both as carbon sources and as toxicants with potential to inhibit yeast growth and fermentation is discussed below. Other toxic compounds are likely present in pectin-rich residues. This is the case for heavy metals that in small amounts are essential micronutrients for yeasts but when they reach toxic concentrations induce the generation of reactive oxygen species (ROS) leading to oxidative stress and loss of biological functions (Mukherjee et al. 2017). The pesticides (fungicides, herbicides and insecticides) used in agriculture may also be present in significant amounts, varying among countries although the maximum residual levels allowed are regulated (European Parliament 2009). Other toxic compounds, for instance phenolic compounds and furans, resulting from acid hydrolysis, may also be present (Berlowska et al. 2018). The accumulation of ethanol or other toxic metabolites are additional sources of combined chemical stresses challenging yeast performance during the bioprocess.

#### Acetic acid and galacturonic acid as carbon sources and toxic compounds

**Fig. 7:**
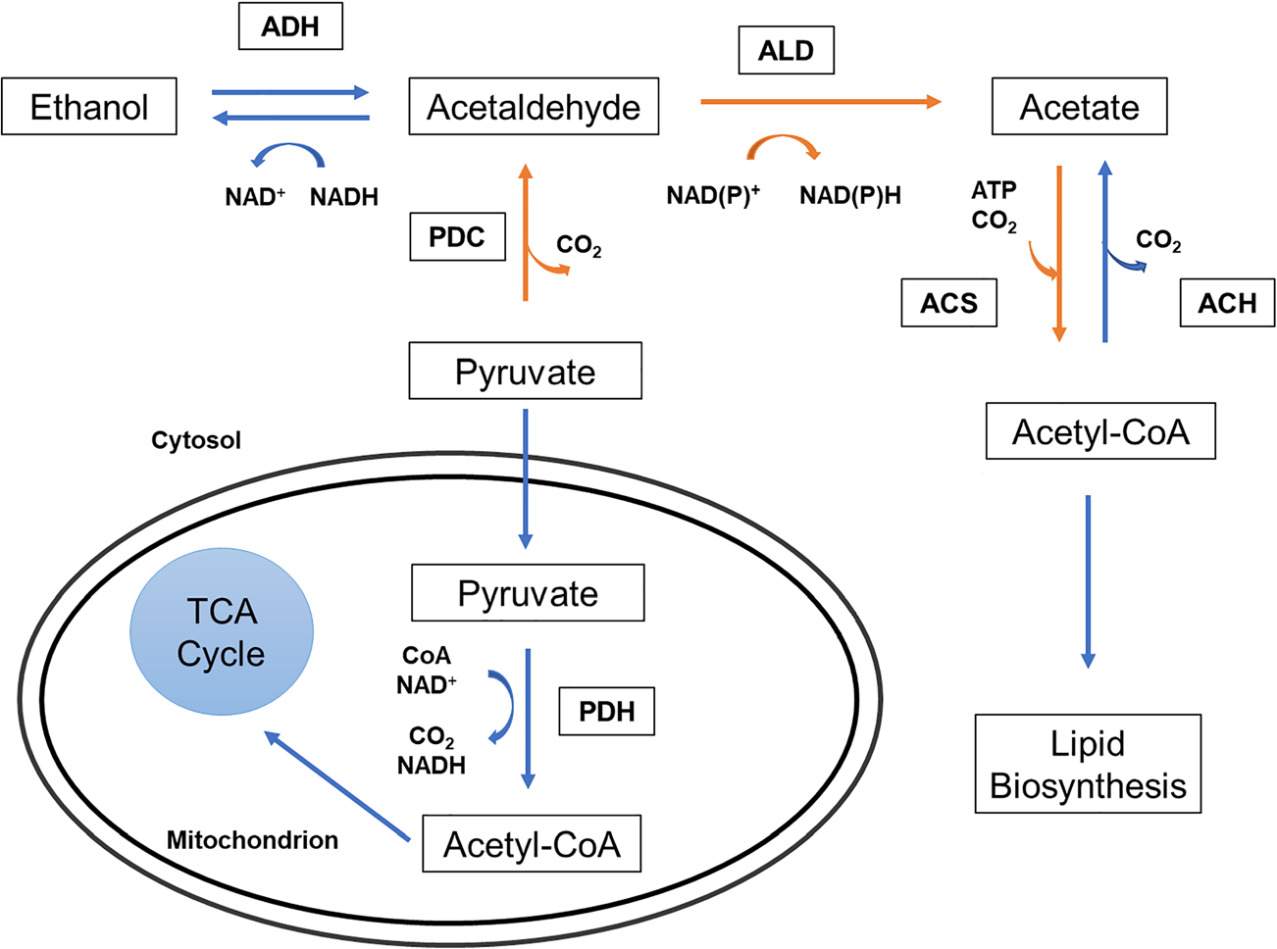
Acetic acid metabolism in yeast. The PDH pathway is indicated by blue arrows, while the PDH bypass is indicated by orange arrows. PDH: pyruvate dehydrogenase; PDC: pyruvate decarboxylase; ALD: aldehyde dehydrogenase; ACS: acetyl-CoA synthetase; ADH: alcohol dehydrogenase (Huang et al. 2016)

Acetic acid is present in pectin-rich residue hydrolysates at higher concentrations in sugar beet pulp hydrolysates compared with citrus peel hydrolysates (Grohmann et al. 1999; Günan Yücel and Aksu 2015), as discussed before. Acetic acid is also a yeast metabolite generated during growth and fermentation. Acetic acid is a source of carbon and energy for a large number of yeasts and can be converted into lipids (Huang et al. 2016) (Fig. 7). Most of the yeast species capable of growing in high acetic acid concentrations are oleaginous, since acetate can be assimilated and converted into acetyl-CoA, a lipid biosynthesis precursor (Spagnuolo et al. 2019). At sub-lethal concentrations, acetic acid is catabolised by several yeast species, like *S. cerevisiae*, *Candida utilis*, *Torulaspora delbruecki* and *Dekkera anomala*, its utilisation being repressed by glucose (Radecka et al. 2015). However, d-glucose and acetic acid are simultaneously catabolised in the highly tolerant *Zygosaccharomyces bailii* species (Rodrigues et al. 2012).

Depending on the level of acetic acid-induced stress and on the tolerance of a specific yeast strain, acetic acid can act as a growth inhibitor due to the ability of the non-dissociated form (pKa 4.7) to diffuse across plasma membrane and cause toxicity when in the cytosol (Mira et al. 2010a, b ; Mira et al. 2011; Palma et al. 2018). The subsequent deprotonation of this acid in the cytosol, with a pH around neutrality, leads to the accumulation of the acetate counter-ion and cytosol acidification (Carmelo et al. 1997). The effect of a specific concentration of acetic acid is particularly drastic at pH below the pKa of the acid. To obtain a holistic view on the toxic effects and the adaptive responses of yeasts to acetic acid, the following review paper is suggested (Palma et al. 2018). The non-conventional food spoilage yeast species *Z. bailii* is able to thrive in acid foods and beverages due to its remarkable tolerance to weak acids at low pH (Mira et al. 2014; Palma et al. 2017). In fact, *Z*. *bailii* is able to grow at concentrations of acetic acid 3-fold higher (370–555 mM) than *S. cerevisiae* (80–150 mM) (Palma et al. 2015; Palma et al. 2018). The remarkable tolerance of *Z. bailii* to weak acids has brought to light the potential of this yeast species as an alternative cell factory for the production of high levels of weak acids (Palma and Sá-Correia 2019). Moreover, the understanding of the mechanisms underlying the tolerance to weak acids in *Z. bailii* sensu *lato* allows the identification of candidate molecular targets for the rational genome engineering for the construction of more robust *S. cerevisiae* strains (Mira et al. 2014; Guerreiro et al. 2016; Palma et al. 2017). Other reported acetic acid-tolerant yeast species are *Pichia kudriavzevii* (Dandi et al. 2013) and *Candida glycerinogenes* (Ji et al. 2016; Zhao et al. 2019). For efficient bioconversion of pectin-rich residues hydrolysates rich in acetic acid, the use of tolerant strains and/or a pH above this weak acid’s pKa is required.

The presence of d-galacturonic acid, even at low concentrations (up to 10 g/L) and pH 3.5 (below the pKa of the acid), in a cultivation medium with a mixture of glucose, galactose, xylose, and arabinose, mimicking pectin-rich residue hydrolysates, was reported to affect the fermentation of most of the sugars with the exception of glucose by a genetically engineered pentose-fermenting strain *S. cerevisiae* CEN.PK 113-7D grown under anaerobiosis (Huisjes et al. 2012). However, at pH 5, at which the concentration of the undissociated toxic form is low, sugar fermentation performance was not affected by the presence of d-galacturonic acid (Huisjes et al. 2012).

#### Methanol as carbon source and toxic compound

Methanol is another toxic compound likely present in pectin-rich hydrolysates. Methanol toxicity mechanisms are poorly studied but, like ethanol and other alcohols, the cell membranes are the anticipated molecular targets (van der Klei et al. 2006). Methanol can be converted into formaldehyde which is a more toxic compound (Yasokawa et al. 2010). For *S. cerevisiae*, 1.23 M of methanol or 1.8 mM of formaldehyde, are concentrations reported to inhibit growth without causing cell death (Yasokawa et al. 2010).

Despite *S. cerevisiae* inability to grow in methanol, there are several non-conventional yeasts that can efficiently use it as the sole carbon and energy source. Since methanol is an inexpensive carbon source, methylotrophic yeasts have been examined for biotechnological applications, ranging from the production of single-cell protein (SCP) and heterologous recombinant proteins to the production of number of chemical compounds (Limtong et al. 2008; Johnson 2013b; Siripong et al. 2018). The most well-known methylotrophic yeast species are *Candida boidinii*, *Ogataea* (*Pichia*) *methanolica*, *Komagataella* (*formerly Pichia*) *pastoris*, *Ogataea minuta* and *Ogataea* (*formerly Hansenula*) *polymorpha*, as well as *Candida parapsilosis*, *Candida* (*formerly Torulopsis*) *glabrata* and *Ogataea* (*formerly Pichia*) *thermomethanolica* (Limtong et al. 2008; Kurtzman and Robnett 2010; Johnson 2013b). The successful genetic modification of *S. cerevisiae* by expressing enzymes from *Pichia pastoris* methanol catabolic pathway (*AOX*, encoding alcohol oxidases, *CAT* encoding a catalase, *DAS*, encoding a dihydroxyacetone synthase, and *DAK*, encoding a dihydroxyacetone kinase) enabled the consumption by the recombinant *S. cerevisiae* strain of 50% of initial methanol concentration (Dai et al. 2017).

#### Heavy metals and agricultural pesticides as toxic compounds

Heavy metals are essential micronutrients for yeasts. However, when above concentration threshold, they induce the generation of reactive oxygen species (ROS) leading to oxidative stress with the oxidation of proteins, lipids and nucleic acids, thus affecting their biological functions (Mukherjee et al. 2017). In general, pectin can bind different heavy metals depending on their structure and natural environment (following preference: Pb^2+^ >> Cu^2+^ > Co^2+^ (cobalt) > Ni^2+^ (nickel) >> Zn^2+^ > Cd^2+^. Sugar beet biomass has preferential affinity for Cu^2+^and Pb^2+^ (Schiewer and Patil 2008). The tolerance to heavy metals is strain-dependent and the variability is large among strains of the same species to different metals (Balsalobre et al. 2003; Vadkertiová and Sláviková 2006). The pesticides (fungicides, herbicides and insecticides) used in agriculture vary among countries, but the maximum residual levels allowed are regulated in the EU and by FDA. The mechanisms of toxicity and tolerance to agricultural pesticides in yeasts are more poorly studied, although the global effects of the herbicide 2,4-D and the agricultural fungicide mancozeb, among others, have been reported (Teixeira et al. 2007; Dias et al. 2010; dos Santos 2012).

### Bioconversion of carbon source mixtures: the challenges

The hydrolysates prepared from pectin-rich residues include a wide range of different carbon sources (C-sources) at variable concentrations, depending on the type of biomass and their processing conditions, as detailed above. The assimilation of usable C-sources by yeasts is strictly regulated and most of the catabolic pathways are subject to CCR (Simpson-Lavy and Kupiec 2019). This constitutes a major challenge for the efficient and economic utilisation of complex substrates in biotechnological processes since in the presence of a preferred sugar, the uptake of secondary carbon sources is inhibited and their sequential utilisation prolong the fermentation time. When d-glucose is present in the extracellular medium, the uptake and catabolism of other carbon sources is repressed in *S. cerevisiae* (Kayikci and Nielsen 2015; Wu et al. 2016; Lane et al. 2018). Strategies for circumventing CCR are especially important when it comes to the use of inexpensive and renewable feedstocks containing mixtures of carbon sources, such as in the case of pectin-rich residues. In fact, the separation of individual substrates is costly and impractical and for this reason, the efficient utilisation of substrate mixtures is a necessity that requires additional strain-improvement efforts (Gao et al. 2019). Efforts to enable C-sources co-utilisation include the introduction of non-native sugar transporters or catabolic pathways that are not subject to CCR or by adaptive evolution and targeted genome engineering (Papapetridis et al. 2018). Yeast strains are susceptible to CCR and in the specific case of pectin-rich biomass hydrolysates, the glucose present is used at first and galactose is expected to be consumed subsequently since the Leloir pathway, through which a molecule of d-galactose is converted into glucose-1-phosphate ready to be used in glycolysis (Sellick et al. 2008), is repressed by glucose (Huisjes et al. 2012). In the case of strains capable of using the other less easily metabolised carbon sources, they will be used sequentially. For example, the strain *M. guilliermondii* FTI 20037 was found to have a native ability to catabolise hexose and pentoses, but when cultivated in a mixed-sugars medium, l-arabinose is only consumed when d-glucose and d-xylose are completely depleted from the medium (Mussatto et al. 2006). However, the simultaneous co-consumption of d-glucose, d-xylose and l-arabinose by *Pseudozyma hubeiensis* IPM1–10 in artificial hydrolysate of lignocellulosic biomass (mixed-sugar medium) was reported leading to the production of high amounts of lipids in less time compared with single-sugar media (Tanimura et al. 2016). Very recently, a pentose-fermenting strain *S. cerevisiae* YE9 expressing the fungal d-galacturonic acid pathway and deleted from *PHO13* and *ALD6* genes (see above) was able to co-consume d-galacturonic acid, l-arabinose and d-xylose (mixed-sugar medium), showing a low susceptibility to catabolic repression (Jeong et al. 2020).

Moreover, d-glucose affects the expression of genes related to other cellular functions such as respiration, gluconeogenesis and the general stress response mechanisms (Lane et al. 2018). The repression of respiration in glucose-containing environments is known as the “Crabtree effect” (Pfeiffer and Morley 2014). The Crabtree effect is observed in *S. cerevisiae* that even under aerobic conditions undergoes alcoholic fermentation when glucose is present at non-limiting concentrations (Pfeiffer and Morley 2014). The fermentative, Crabtree-positive yeasts include the genera *Saccharomyces*, *Zygosaccharomyces*, *Dekkera* and *Schizosaccharomyces* while Crabtree-negative yeasts include strains belonging to the genera *Pichia*, *Debaryomyces*, *Candida* or *Kluyveromyces* (Rozpędowska et al. 2011).

It is important to notice that the use of recombinant yeasts, constructed based on the application of metabolic engineering and synthetic biology tools, has shown that when single substrates are used, several limitations to their metabolism may occur, resulting in low yield (Liu et al. 2020). For instance, when the target product has distinct chemical properties or requires long synthetic routes from starting substrates (Babel 2009). The improvement of product biosynthesis through the optimal balance of biosynthetic components can be achieved by the application of mixed substrates, changing flux distribution and cellular resources, instead of intensive genetic modifications (Liu et al. 2020).

### Value-added bioproducts from pectin-rich hydrolysates by non-conventional yeasts

**Fig. 8:**
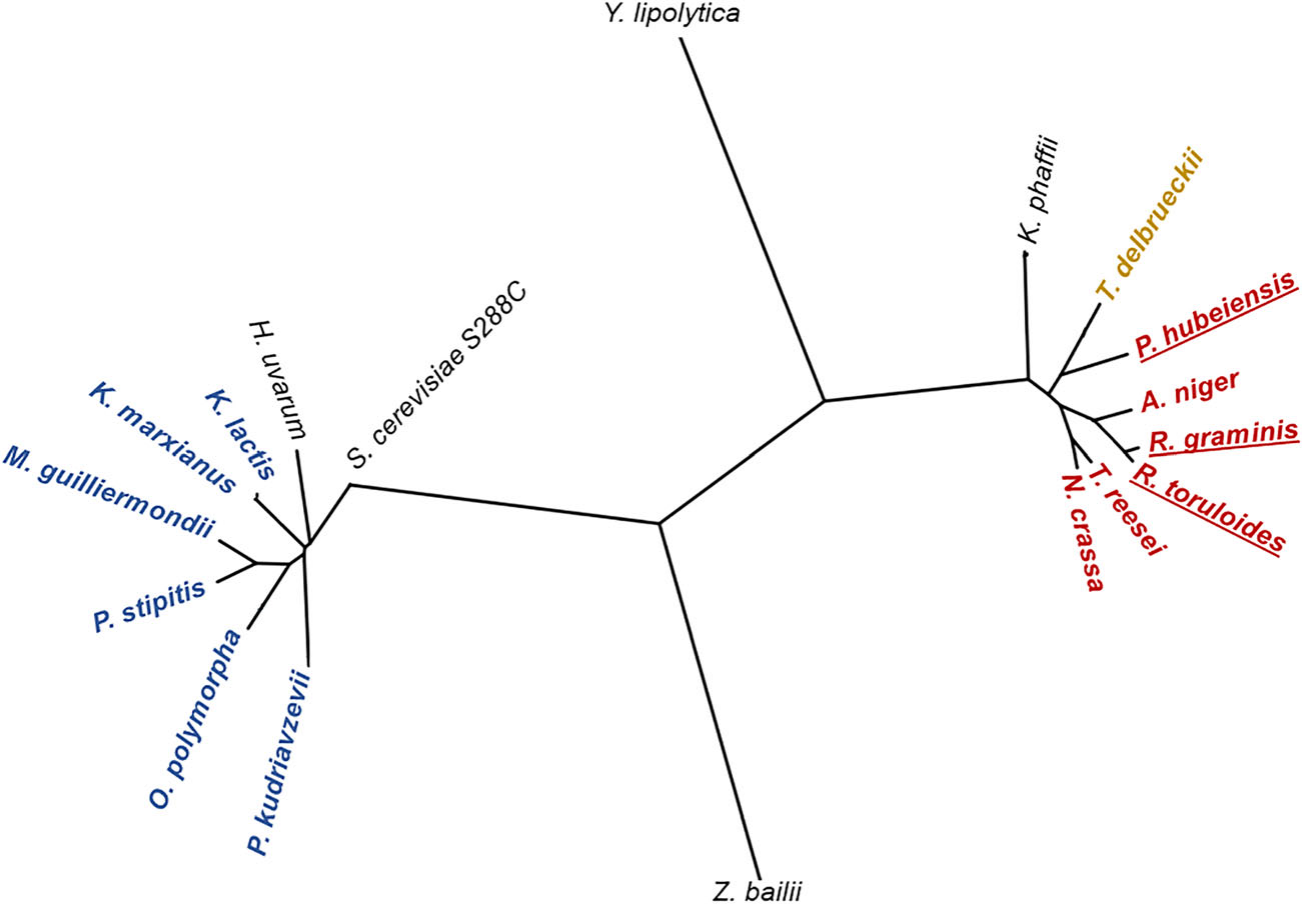
Phylogenetic tree of relevant yeasts and related filamentous fungi discussed in this work. The tree was constructed using the neighbour-joining method based on the alignment of the large subunit (26S) ribosomal DNA sequence. The sequences used were obtained from “EnsemblFungi” database. The yeasts coloured with blue (the Ascomycetous yeasts *Kluyveromyces marxianus*, *Kluyveromyces lactis*, *Meyerozyma guilliermondii*, *Pichia stipitis*, *Ogataea polymorpha* and *Pichia kudriavzevii*) are capable of utilizing d-xylose and l-arabinose as carbon sources (C-sources). Red colour represented basidiomycetous yeasts (underlined), such as *Rhodosporidium toruloides*, *Rhodotorula graminis* and *Pseudozyma hubeiensis* and filamentous fungi (*Trichoderma reesei*, *Aspergillus niger* and *Neurospora crassa*) which are able to grow in d-galacturonic acid and also in d-xylose and l-arabinose. The yeast species *Torulaspora delbrueckii* represented in yellow is capable to grow in d-galacturonic acid and d-xylose. The phylogenetic tree also includes (black colour) *Saccharomyces cerevisiae* S288C, *Zygosaccharomyces bailii*, *Yarrowia lipolytica* and *Komagataella phaffii*. The yeast species *S. cerevisiae K. marxianus*, *M. guilliermondii*, *P. stipitis*, *P. kudriavzevii* and *T. delbrueckii* are interesting bioethanol producers, while *H. uvarum* is also responsible for the fruity-like aromatic compounds in fermented beverages. *Y. lipolytica*, *P. hubeiensis*, *R. graminis* and *R. toruloides* are oleaginous yeasts which can convert C-sources into high concentrations and a wide range of lipids. The species *K. phaffii* is mainly used as cell factory for heterologous protein expression while *Z. bailii* exhibits a remarkable tolerance to weak acids

The interest in non-*Saccharomyces* yeasts is gaining momentum due to that a variety of important features they possess that are not present in the model yeast *S. cerevisiae* making this large group of yeast species/strains desirable cell factories for the synthesis of a wide range of added-value products (Radecka et al. 2015). These traits of metabolic versatility and yeast physiology are highly valuable for the biosynthesis of interesting added-value compounds from pectin-rich residue hydrolysates (Wagner and Alper 2016; Rebello et al. 2018). A significant genetic distance is observed in the phylogenetic tree prepared for yeasts exhibiting different capacities to catabolise pentoses (among the ascomycetous yeasts) or d-galacturonic acid (basidiomycetous yeasts, close to filamentous fungi with a similar metabolic trait) (Fig. 8).

Although several non-conventional yeasts with potential for the bioconversion of pectin-rich wastes have received the “generally recognised as safe” (GRAS) label from FDA (Food and Drug Administration), there are several interesting species that are, unfortunately, reported opportunistic pathogens (Wirth and Goldani 2012; Johnson 2013a). The genera *Cryptococcus*, *Candida* and *Rhodotorula* are some of those encompassing pathogenic species, such as *Cryptococcus neoformans* and *Cryptococcus gatii* (Johnson 2013a) as well as *Rhodotorula mucilaginosa*, *Rhodotorula glutinis* and *Rhodotorula minuta* (Wirth and Goldani 2012). However, the potential of some of them to produce interesting metabolites is high, and therefore, they are potential sources of genetic information for the engineering of GRAS species.

There is a wide range of products synthesised by different non-conventional yeast species using pectin-rich substrates that have been reported in the literature. A summary of these examples is shown in Table 3. Most of the bioethanol in the market is produced from hexose fermentation by the yeast *S. cerevisiae*, namely from glucose and fructose. However, several yeast species are also able to ferment other sugars present in pectin-rich residues and were reported as bioethanol producers from that biomass. This is the case for strains of *K. marxianus* (Serrat et al. 2004), *M. guilliermondii* (Schirmer-Michel et al. 2008; Schirmer-Michel et al. 2009), *Scheffersomyces* (*Pichia*) *stipitis* (Günan Yücel and Aksu 2015), *P. kudriavzevii* (Kaur Sandhu et al. 2012), *Hanseniaspora uvarum* and *Hanseniaspora valbyensis* (Rodríguez Madrera et al. 2015).

**Table 3:** Reported examples of bioconversion of pectin-rich residues by non-conventional yeasts

Yeast	Pectin-rich residues	Initial sugar concentration (of total sugars in hydrolysate)	Bioproducts (final concentration or yield)	References
*Scheffersomyces* (*Pichia*) *stipitis* NRRL Y-7124	Sugar beet pulp hydrolysate	75 g/L	Ethanol (37.1 g/L)	Günan Yücel and Aksu (2015)
*Pichia kudriavzevii* KVMP10	Kinnow mandarin peels hydrolysate	79 g/L	Ethanol (34 g/L)	Kaur Sandhu et al. (2012)
	Orange peel hydrolysate	101 g/L	Ethanol (54 g/L)	Koutinas et al. (2016)
*Candida parapsilosis* IFM 48375	Orange peel hydrolysate	–	Ethanol (0.85 g EtOH/4.2 g of dry matter of orange peel)	Tsukamoto et al. (2013)
*Candida parapsilosis* NRRL Y-12969	Orange peel hydrolysate	–	Ethanol (0.76 g EtOH/4.2 of dry matter of orange peel)	Tsukamoto et al. (2013)
*Hanseniaspora uvarum* H.u. 283	Apple pomace hydrolysate	36 g/kg of apple pomace	Ethanol (2.8% (w/w) of reducing sugars) Volatile fruity-like aroma compounds (esters and γ-Nonalactone)	Rodríguez Madrera et al. (2015)
*Hanseniaspora valbyensis* H.v. 43	Apple pomace hydrolysate	36 g/Kg of apple pomace	Ethanol (2.8% (w/w) of reducing sugars) Volatile fruity-like aroma compounds (esters and γ-Nonalactone)	Rodríguez Madrera et al. (2015)
*Yarrowia lipolytica* MYA-2613	Apple pomace hydrolysate	80 g/L	Lipids (25.8 g/L (C16:0; C18:0; C18:1 C20:0)	Liu et al. (2019)
*Trichosporon cutaneum* AS 2.571	Beet pulp hydrolysate	52 g/L	Lipids (7.2 g/L) (palmitic; stearic; oleic, linolenic)	Wang et al. (2015)
*Trichosporon fermentans* CICC 1368	Beet pulp hydrolysate	52 g/L	Lipids (5.8 g/L) (palmitic; stearic; oleic, linolenic)	Wang et al. (2015)
*Cryptococcus curvatus* ATCC 20509	Beet pulp hydrolysate	52 g/L	Lipids (6.9 g/L) (palmitic; stearic; oleic, linolenic)	Wang et al. (2015)
*Rhodosporidium toruloides NRRL1091*	Orange peel waste	18 g/L	Lipids (5.8 g/L) (palmitic; oleic)	Carota et al. (2020)
*Cryptococcus laurentii UCD 68–201*	Orange peel waste	18 g/L	Lipids (4.5 g/L) (palmitic; oleic)	
*Rhodotorula* sp.	Apple pomace hydrolysate	40 g/L	Carotenoids (16.8 mg/100 g DCW)	Joshi et al. (2013)
*Trichosporon penicillatum* SNO-3	Citrus peel hydrolysate (*Citrus unshiu*)	23.2% (w/w)	Protopectin-solubilizing enzyme	Sakai and Okushima (1980)
*Torula* (*Candida*) *utilis* CCT3469	Apple pomace hydrolysate	15%(w/w)	Lignocellulosic enzymes: pectinase (25 μg/mL), manganese-dependent peroxidase (2.5 μg/mL), cellulase and xylanase (< 1 μg/mL)	Villas-Bôas et al. (2002)
*Torula* (*Candida*) *utilis* DSM 70163	Sugar beet pulp hydrolysate	45 g/L	Single cell protein (43% g protein/g sugar consumed)	Nigam and Vogel (1991)
*Candida tropicalis* DSM 7015		45 g/L	Single cell protein (39% g protein/g sugar consumed)	Nigam and Vogel (1991)
*Candida parapsilosis* DSM 70125		45 g/L	Single cell protein (34% g protein/g sugar consumed)	Nigam and Vogel (1991)
*Candida solani* ATCC 14440		45 g/L	Single cell protein (35% g protein/g sugar consumed)	Nigam and Vogel (1991)

*H. uvarum* and *H. valbyensis* strains produce volatile fruity-like aroma compounds, with high acetic acid ester content, from apple pomace (Rodríguez Madrera et al. 2015). These volatile or non-volatile aromatic compounds are very valuable ingredients in chemical, food, cosmetic and pharmaceutical industries (Martínez et al. 2017) and comprise 25% (aroma compounds) of global market of food additives (Rodríguez Madrera et al. 2015).

Single-cell oil and lipids, namely fatty acids, are obtained from oleaginous yeasts, for utilisation as substitutes for vegetable oils and animal and vegetal fats (e.g. as cocoa butter) (Wang et al. 2012). The demand for biobased-fuels to replace fossil-based-products has led to an increase of biodiesel production and other oleochemical products from oleaginous yeasts (Wang et al. 2012; Anschau 2017). Yeast species, such as *Y. lipolytica*, *Trichosporon cutaneum*, *Trichosporon fermentans* and *Cryptococcus curvatus*, were reported as yeast platforms to produce different levels of fatty acids from pectin residues. Remarkably, *C. curvatus* can convert acetate (5 g/L, at pH 6.0) into oils (up to 50% (w/w) of lipid accumulation in the biomass) (Christophe et al. 2012) and *Rhodosporidium toruloides* can convert 20 g/L of acetic acid (at pH 6.0) in lipids up to 48% (w/w) of the biomass (Huang et al. 2016). *R. toruloides* lipids are mainly triacylglycerols (C_16_ and C_18_ fatty acids) (Singh et al. 2018) and the dried cellular biomass can be directly converted into biodiesel (Guo et al. 2019). A recent study conducted with 18 strains of oleaginous yeasts also reported the accumulation of lipids in *R. toruloides* NRRL 1091 and *Cryptococcus laurentii* UCD 68-201 (77 and 47% on a dry matter basis, respectively) from orange peel extract (Carota et al. 2020).

A *Rhodotorula sp.* strain, isolated from spoiled sauerkraut, was reported to grow and produce carotenoids from 50 g/L apple pomace, but the addition of 0.3% (v/v) ferrous ammonium sulphate led to the highest carotenoid concentration (Joshi et al. 2013). Oleaginous yeasts were identified as capable of growing in medium containing only d-galacturonic acid as carbon source. This is the case of the species *C. laurentii*, *C. curvatus*, *Cryptococcus* cf. *aureus*, *Cryptococcus ramirezgomezianus*, *Leucosporidiella creatinivora*, *Tremella encephala*, *Geotrichum fermentans*, *R. mucilaginosa*, *Trichosporon dermatis* and *Trigonopsis variabilis* which exhibit relevant genetic information related with d-galacturonic acid metabolic pathway for alternative expression in *S. cerevisiae* (Sitepu et al. 2014).

The production of enzymes from agro-industrial residues by yeasts is still one of the most relevant applications for these substrates, in particular for the production of pectinases (Vendruscolo et al. 2008). Single-cell protein (SCP) or yeast components can easily be produced from several agro-industrial wastes and are extremely useful for food and feed nutritional enrichment (Vendruscolo et al. 2008; Johnson and Echavarri-Erasun 2011). *Torula utilis*, *Candida tropicalis*, *Candida parapsilosis* and *Candida solani* are sources of SCP from sugar beet pulp (Nigam and Vogel 1991).

### Metabolic engineering of non-conventional yeasts with potential for the bioconversion of pectin-rich residues

The application of metabolic engineering strategies to non-conventional yeasts envisages the resolution of the problems discussed in previous sections, in particular the co-utilisation of different carbon sources, the enhancement of the tolerance to the inhibitors commonly present in the hydrolysates and other bioprocess-related stresses and the improvement of, or the production of, novel bioproducts. However, the metabolic engineering of non-conventional yeasts faces several challenges such as the reduced availability of stable and high copy number of plasmids and suitable approaches for foreign DNA integration into the host’s genome (Löbs et al. 2017). For industrial bioprocesses, metabolic engineering requires genomic integration of genetic information for high stability of the expression cassette over extended cultivations, homogenous expression levels in cell population and the elimination of the selective marker (Löbs et al. 2017). Currently, there are several genome editing tools already available for metabolic engineering of non-conventional yeasts (Gupta and Shukla 2017). The CRISPR technology is allowing gene disruptions and integrations in several yeast species, such as *Kluyveromyces lactis*, *K. marxianus*, *S. stipitis*, *Y. lipolytica*, *Hansenula polymorpha* and *P. pastoris* (Weninger et al. 2015; Gao et al. 2016; Löbs et al. 2017; Raschmanová et al. 2018; Nurcholis et al. 2020). The perspectives of the metabolic engineering of non-conventional yeasts more suited to industrial bioprocesses are encouraging, supported by the increased availability of genome sequences obtained by next-generation sequencing and the development and availability of genome editing and bioinformatic tools. Among them is the YEASTRACT+ database that also provides biological information and tools for the analysis and prediction of transcription regulatory associations at the gene and genomic levels in non-conventional yeasts of biotechnological interest, in particular *Z. baillii*, *K. lactis*, *K. marxianus*, *Y. lipolytica* and *K. phaffii* (Monteiro et al. 2020)*.* These developments are paradigmatic examples that the exploitation of non-*Saccharomyces* yeasts is gaining momentum.

From the already significant number of examples of metabolic engineering of yeasts for biomass bioconversion, only a few examples of potential interest for the bioconversion of pectin-rich biomass were reported. Oleaginous yeasts are being intensively studied due to their native mechanisms to convert carbon sources into neutral lipids and lipid-derived compounds. For example, the triacylglyceride pathway was engineered into *Y. lypolytica* by introducing a synthetic pathway that enhances glycolysis activity with an improvement in glycolytic NADH and an increase of approximately 25% of lipid biosynthesis from glucose (Qiao et al. 2017). Tools for the genetic engineering of the oleaginous yeast species *R. toruloides* to improve the production of carotenoids and lipids were recently developed (Park et al. 2018). The metabolic engineering of *K. lactis* by the construction of a null mutant in a single gene encoding a mitochondrial alternative internal dehydrogenase led to a metabolic shift from respiration to fermentation, increasing the rate of ethanol production (González-Siso et al. 2015). In *K. marxianus*, the simultaneous knockdown of the TCA cycle and the electron transport chain genes *ACO2b*, *SDH2*, *RIP1* and *MSS51*, resulted in a 3.8-fold increase in ethyl acetate productivity from glucose (Löbs et al. 2018). The examples of genetic manipulation of non-conventional yeasts for sugar transporters are not many, but the heterologous integration of the xylose transporter gene *AT5G17010* from *A. thaliana* into *C. tropicalis* resulted in a 37–73% increase in xylose uptake compared to the original strain (Jeon et al. 2013). Given that synthetic biology methods and tools are being adapted to be used in non-conventional yeasts, the construction of engineered strains with specific traits for the more efficient bioconversion of pectin-rich agro-industrial residues can be anticipated.

## Concluding remarks

The valorisation of pectin-rich residues resulting from the industrial processing of fruits and vegetables for the production of value-added compounds by non-conventional yeast species is gaining momentum. The challenges posed to the industrial implementation of efficient bioprocesses are however many and thoroughly discussed in this review paper. The challenges encountered, at the biological level, range from the simultaneous effective metabolisation of C-source mixtures present in pectin-rich residue hydrolysates and the required increase of yeast robustness to cope with the multiple potential stresses encountered during specific bioprocesses, to the improvement of production of interesting and novel metabolites.
